# Spatial coefficient of variation of arterial spin labeling MRI as a cerebrovascular correlate of carotid occlusive disease

**DOI:** 10.1371/journal.pone.0229444

**Published:** 2020-02-26

**Authors:** Henri J. M. M. Mutsaerts, Jan Petr, Reinoud P. H. Bokkers, Ronald M. Lazar, Randolph S. Marshall, Iris Asllani

**Affiliations:** 1 Department of Radiology and Nuclear Medicine, Amsterdam University Medical Center, Location VUMC, Amsterdam, The Netherlands; 2 Department of Biomedical Engineering, Institute Hall, Rochester Institute of Technology (RIT), Rochester, New York, NY, United States of America; 3 Helmholtz-Zentrum Dresden-Rossendorf, Institute of Radiopharmaceutical Cancer Research, Dresden, Germany; 4 Department of Radiology, Medical Imaging Center, University Medical Center Groningen, University of Groningen, Groningen, Netherlands; 5 Department of Neurology, UAB, McKnight Brain Institute, University of Alabama at Birmingham, Birmingham, AL, United States of America; 6 Department of Neurology, Columbia University Irving Medical Center, New York, NY, United States of America; 7 Clinical Imaging Sciences Centre, Neuroscience, University of Sussex, Brighton, United Kingdom; Massachusetts General Hospital, UNITED STATES

## Abstract

Clinical interpretation of arterial spin labeling (ASL) perfusion MRI in cerebrovascular disease remains challenging mainly because of the method’s sensitivity to concomitant contributions from both intravascular and tissue compartments. While acquisition of multi-delay images can differentiate between the two contributions, the prolonged acquisition is prone to artifacts and not practical for clinical applications. Here, the utility of the spatial coefficient of variation (sCoV) of a single-delay ASL image as a marker of the intravascular contribution was evaluated by testing the hypothesis that sCoV can detect the effects of differences in label arrival times between ipsi- and contra-lateral hemispheres even in the absence of a hemispheric difference in CBF. Hemispheric lateralization values for sCoV and CBF were computed from ASL images acquired on 28 patients (age 73.9 ± 10.2 years, 8 women) with asymptomatic unilateral carotid occlusion. The results showed that sCoV lateralization predicted the occluded side with 96.4% sensitivity, missing only 1 patient. In contrast, the sensitivity of the CBF lateralization was 71.4%, with 8 patients showing no difference in CBF between hemispheres. The findings demonstrate the potential clinical utility of sCoV as a cerebrovascular correlate of large vessel disease. Using sCoV in tandem with CBF, vascular information can be obtained in image processing without the need for additional scan-time.

## Introduction

Patients with asymptomatic carotid steno-occlusive pathology are at increased risk for irreversible ischemia, particularly when there is cerebral hemodynamic impairment in the territory of the obstructed carotid [1,2]. While effective endovascular and other interventions are available for those with high grade stenosis, they come with substantial procedural risks of stroke and death [3]. Therefore, a comprehensive risk-benefit assessment at the single-patient level is essential for identifying patients who are asymptomatic but who nevertheless may be at high risk of stroke and thus would benefit from endovascular treatment. Reliable, non-invasive, and sensitive neuroimaging markers can greatly improve the efficacy of such an assessment [4]. Cerebral blood flow (CBF) measured with arterial spin labeling (ASL) MRI [5] meets the criteria and has the potential to be used for simultaneously evaluating vascular insufficiency/reconfiguration and presence of collateral flow. This clinical potential of ASL remains largely untapped [6,7].

While clinical applications of ASL are on the rise, interpretation of CBF ASL images continues to be hampered by the uncertainty in whether the signal originates from tissue or macro-vasculature. This complication is especially relevant in diseases such as carotid steno-occlusive where hemodynamic changes are regionally dependent. In these cases, ASL acquired with varying post-labeling delays is used to estimate the arterial transit time (ATT), which is defined as the time the blood travels from the labeling plane to the microvasculature within an imaged voxel [8,9]. Multi-PLD ASL can thus provide a multi-parametric assessment of the hemodynamic status, which reflects local reconfiguration of perfusion pathways and may include changes in blood volume, flow, and ATT [10]. In the past, single-PLD ASL was preferred to multi-PLD due to issues with SNR, motion artifacts, and increased scan-time, all of which are particularly relevant when scanning vascularly compromised patients [11–13]. With improvements in ASL sequences and expanded availability of 3T scanners, multi-PLD ASL is now increasingly used in clinical research [14,15]. However, following the consensus recommendation for clinical ASL [5], most clinical studies continue to use the single-PLD implementation of ASL.

Spatial coefficient of variation (sCoV) of the ASL signal in a single PLD image has been proposed as a proxy parameter of ATT [16]. The rationale follows from the assumption that delayed intra-vascular label is reflected in the spatial variance of the ASL signal [17]. Here, as proof-of-principle, we investigated the potential utility of the sCoV as a marker of vascular health in asymptomatic patients with unilateral carotid occlusion. Because sCoV is not dependent on complete arrival of label in the tissue, it may make ASL more clinically feasible in diseases with prolonged ATT. As such, carotid occlusive patients provide a unique opportunity for clinical validation of sCoV due to of the gradual collateralization and vascular reconfiguration that occur in the occluded hemisphere [18]. These changes are expected to feature a different compartmental distribution—intravascular *vs*. tissue—of the ASL signal compared to the contra-lateral hemisphere, where the intravascular contribution is expected to be lower due to a more efficient label delivery into the tissue [11].

The selection of asymptomatic patients was a key element of the study design, and it was based on the assumption that asymptomatic patients would have asymmetrical sCoV and close to symmetrical CBF, in contrast to symptomatic patients for whom both sCoV and CBF are likely to be asymmetrical [19]. The central hypothesis of the study was that the asymmetry of the intravascular *vs*. tissue label distribution–i.e., the lateralization of sCoV − is a better predictor of carotid occlusion than the lateralization of the mean CBF.

## Materials and methods

### Patients

Patients were recruited over a period of 2 years through the outpatient practices of the Vascular Neurology group at Columbia University. Patients with an asymptomatic unilateral internal carotid artery (ICA) steno-occlusive lesion (n = 28, 20 men, mean age 73.9 ± 10.2 y) were included if they had either a high-grade stenosis (≥ 80%) or a complete occlusion. From the 28 patients, 17 had the left carotid occluded. The stenosis grade was determined by flow velocities in the internal carotid artery (ICA) at the carotid bifurcation measured with Doppler ultrasound. Patients lacking clinical symptoms, including presence of transient ischemic attack without stroke symptoms, were defined as “asymptomatic” [20]. Exclusion criteria included: prior clinical stroke, diagnosis of dementia, history of head trauma, current substance abuse, major psychiatric disease, NYHA Stage 3/4 congestive heart disease, or contraindication to MRI. The study was approved by the Institutional Review Board of Columbia University Medical Center and all patients provided written informed consent.

### MRI acquisition

Imaging was performed on a 3T system (Achieva, Philips) equipped with a 32-channel head coil. Background-suppressed, single-PLD, 2D-EPI pseudo-continuous ASL (pCASL) images were acquired with: an initial PLD of 1200 ms, PLD range for all slices 1200–2025 ms (accounting for the acquisition of 12 slices in ascending order with acquisition time = 75 ms/slice), labeling duration = 1950 ms, TE/TR = 14/4000 ms, voxel size = 3.75 x 3.75 x 8 mm^3^. The ASL images were acquired with a finger-tapping task with a block design of 4 OFF and 4 ON alternating blocks (each block 2 min long and containing 15 control/label pairs). For this study, only the scans of 4 OFF blocks (in total 60 control/label ASL pairs) were used. A 3D T1-weighted image with spatial resolution 0.83 x 0.83 x 0.9 mm^3^ was acquired for tissue segmentation and registration purposes.

### Image processing & data analysis

ASL images were processed using the automated image processing toolbox ExploreASL [21], which included: motion-correction; segmentation of the T1w image into gray (GM) and white matter partial volume maps using CAT12 [22]; co-registration of the ASL and T1w images; and quantification of CBF using the single compartment model accounting for the PLD difference between slices [5]. Due to the length of the MRI protocol, there was no M0 scan acquired, therefore a previously obtained M0 value was used as a single whole-brain value for the CBF quantification in all patients [23,24]. The anterior cerebral (ACA) and middle cerebral artery (MCA) regions from an MNI vascular territory atlas [25] were combined into left and right ICA anterior vascular territory regions-of-interest (ROIs). The ICA ROIs were subsequently masked using patient-specific GM masks (including only voxels with GM volume >70%). The resulting left and right ROIs were used for the computation of mean CBF and sCoV from the quantified CBF maps. The sCoV was calculated as:

$$sCoV=\frac{\sigma_{CBF}}{\mu_{CBF}}$$ [1]

where σ and μ represent the standard deviation and the mean CBF within the ROI, respectively. For both CBF and sCoV, the asymmetry index (AI) was calculated as the hemispheric difference (left vs right) normalized by the mean:

$$AI=\frac{\left({ASL}_{Left}-{ASL}_{Right}\right)}{0.5\times ({ASL}_{Left}+{ASL}_{Right})}$$ [2]

where CBF or sCoV can be used in place of ASL. Additionally, the AI was computed also between the hemispheres with the occluded and the unoccluded ICAs.

To test whether there was a significant lateralization, we performed Wilcoxon sign test on sCoV and CBF data with level of significance set at *p* < 0.05. We evaluated if the laterality was associated with the side of the occlusion (AI of CBF or sCoV being negative or positive on the occluded side, respectively). To ensure that the observed asymmetries were not potentially due to any ASL sequence effects or differences in GM volume between the ipsi- and contra-lateral hemispheres, the AI was also computed for the GM partial volume maps and the raw ASL control images.

## Results

**Fig 1:**
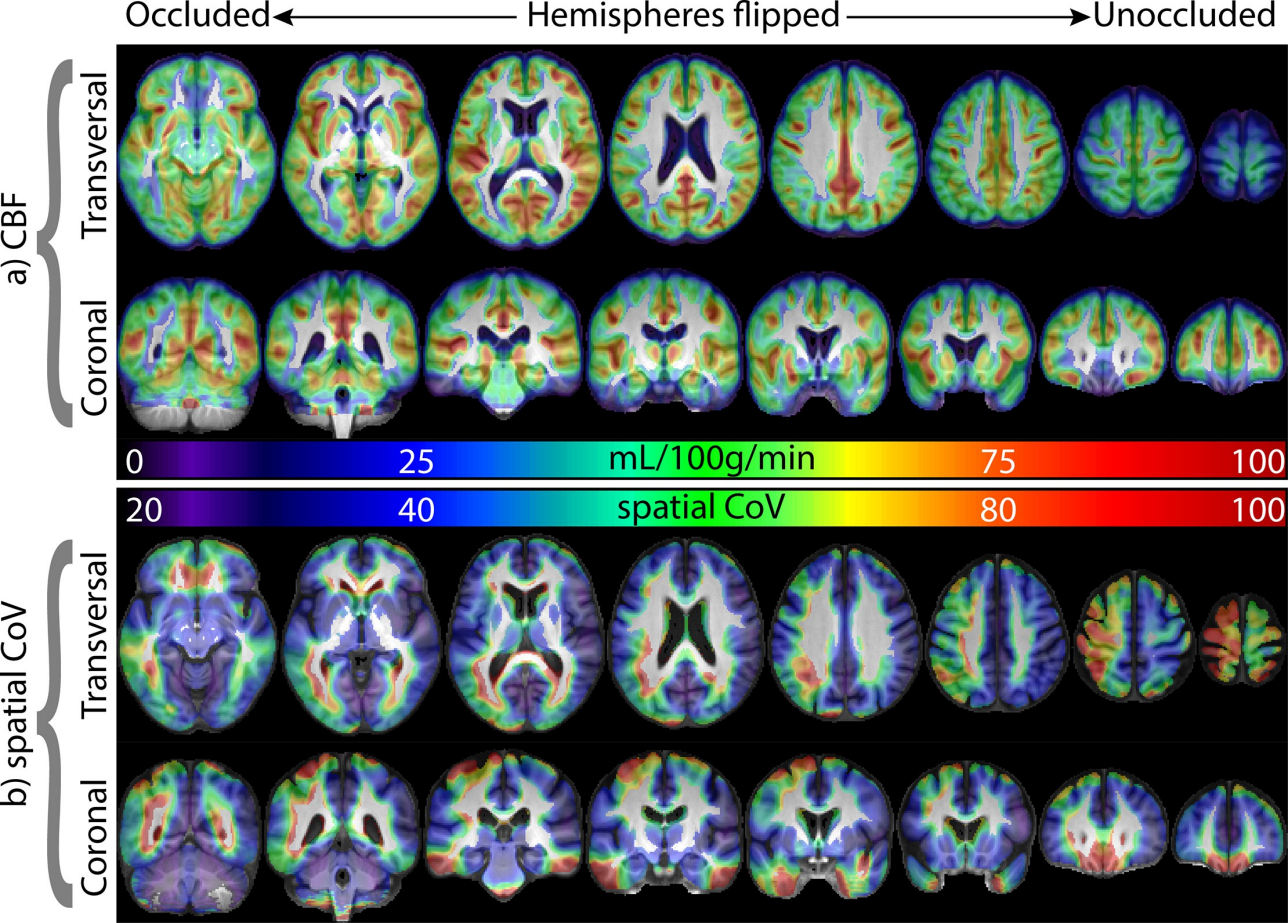
The group average GM CBF and sCoV images are overlaid in color on the grayscale average T1-weighted image. The hemispheres were flipped such that the occluded and unoccluded hemispheres were on the left and right side, respectively, for each participant. Note that lateralization is much clearer for the sCoV than for CBF. Note that for this figure, the sCoV was calculated within an 8x8x8mm^3^ Gaussian neighborhood of each voxel. This was done for visualization purposes only instead of providing a single value of sCoV for the whole hemisphere. The lower sCoV values in the superior regions near the edge of the brain are likely due to higher noise in the ASL signal and partial volume effects in these regions. It is important to emphasize that this had no bearing on the results as sCoV was computed over the entire hemisphere, (Eq 1). GM = gray matter, CBF = cerebral blood flow, sCoV = spatial coefficient of variation.

All patients had stenosis of the “occluded side” of [80–100]%. In the “unoccluded” side, the stenosis ranged from 0 to 60%, (n = 1 with no atherosclerotic plaque; n = 22 with plaque but [1–40]% stenosis; and n = 5 with [41–60]% stenosis). The ICA CBF in the occluded hemisphere was lower than in the unoccluded (57.7 ± 17.1 vs. 60.9 ± 15.9 mL/100g/min, *p* = 0.0357, Fig 1A). In contrast to CBF, the sCoV was higher in the occluded hemisphere than in the unoccluded (48.8 ± 17.3% vs. 39.1 ± 7.7%, *p* = 2*10^−7^, Fig 1B).

**Fig 2:**
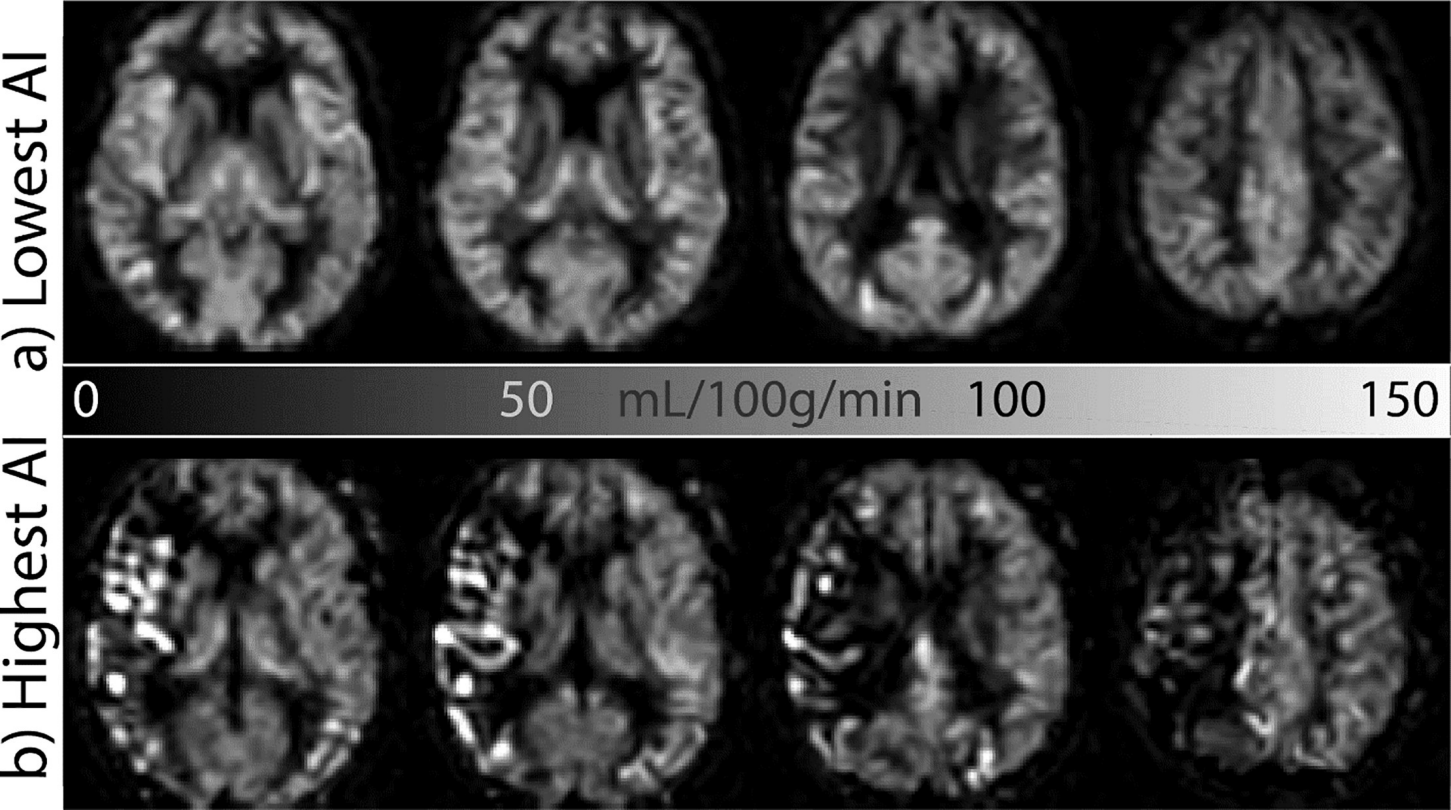
CBF images from the patients with the lowest (a) and highest (b) asymmetry index (AI) of the spatial coefficient of variation, sCoV. Compare the overall symmetric and homogeneous appearance of the gray matter perfusion of (a) with the asymmetric, heterogeneous, vascular appearance of the apparent gray matter perfusion of (b). CBF = cerebral blood flow, sCoV = spatial coefficient of variation.

To provide a visual sense of the macrovascular effects on CBF images from which sCoV was computed, ASL images from patients with highest and lowest spatial variability are shown in Fig 2. The AI between the occluded and the unoccluded hemispheres was different from zero for sCoV but not for CBF (*p* = 0.0002 and *p* = 0.087, respectively). When the AI was computed between left and right hemispheres, without regard for the side of occlusion, no systematic lateralization was found for either CBF (*p* = 0.18) or sCoV (*p* = 0.34). A summary of the results is provided in Table 1.

**Table 1:** Summary of the results for CBF and sCoV across patients (n = 28).

	CBF (mL/100g/min)	sCoV (%)
Left ICA	59.8 ± 16.1	43.0 ± 11.3
Right ICA	58.9 ± 17.0	44.9 ± 16.7
Occluded side	57.7 ± 17.1	48.8 ± 17.3
Non-occluded (stenosed) side	60.9 ± 15.9	39.1 ± 7.7
AI occluded vs non-occluded	-6.5% ± 10.2%	18.2% ± 20.2%*
AI left versus right	2.1% ± 11.9%	-1.5% ± 27.4%

AI = asymmetry index (expressed in percentages), CBF = cerebral blood flow, sCoV = spatial coefficient of variation, ICA = internal carotid artery.

*p<0.001

For 22 patients, the AI of sCoV was above 5%, out of which, 17 had the AI above 10%. There were 2 patients for whom the sCoV AI was under 2%, and only 1 patient for whom the sCoV AI had no predictive value.

The sCoV lateralization predicted the occluded side in all but 1 patient (96.4%). The mean CBF lateralization was associated with the occluded side in 20 out of 28 patients (71.4%; Table 2). There were eight patients for whom spatial CoV, in contrast to CBF, was able to correctly predict the occlusion side (Table 2). For only one patient, the converse was true: CBF was higher in the contralateral hemisphere (106.3 vs 98.2 mL/100g/min) whereas sCoV was essentially the same for both, (36.3% vs 36.0%). The results could not be explained by GM volume or inhomogeneity bias of the ASL sequence as there were no significant lateralization effects in the GM partial volume maps or in the raw ASL control images. For the GM partial volume maps, the AI across patients was 0.48% ± 0.43% for the mean and 0.12% ± 0.6% for the sCoV. For the raw ASL control images, the average AI of the mean signal intensities and sCoV was 0.5% ± 4% and -0.7% ± 5.2%, respectively.

**Table 2:** Performance of CBF and sCoV in predicting the side of occlusion across patients (n = 28).

	Left ICA occlusion (n)	Right ICA occlusion (n)	
Lower ipsilateral ICA CBF (n)	11	9	20
Lower contralateral ICA CBF (n)	6	2	8
	17	11	28
	**Predictive value = 20/28 = 71.4%**		
	Left ICA occlusion (n)	Right ICA occlusion (n)	
Higher ipsilateral ICA sCoV (n)	17	10	27
Higher contralateral ICA sCoV (n)	0	1	1
	17	11	28
	**Predictive value = 27/28 = 96.4%**		

CBF = cerebral blood flow, sCoV = spatial coefficient of variation, ICA = internal carotid artery

## Discussion

The primary finding of this study was that sCoV had higher sensitivity than CBF in predicting the side of carotid occlusion (96.4% vs 71.4%). The sCoV asymmetry associated with occlusion was present in all but 1 patient. In contrast, 8 out of 28 patients were missed using the asymmetry of mean CBF. While predicting the side of occlusion in and of itself has limited clinical value, these findings suggest that sCoV in combination with CBF may extend the clinical utility of ASL as a cerebrovascular marker in carotid occlusive disease. Concomitant use of sCoV may also prove useful in assessing the degree of collateralization, where a higher sCoV may indicate a better collateral score, which subsequently can be included in screening for intracranial and extracranial stenosis. It is important to emphasize, however, that the sCoV alone cannot differentiate to what extent the observed asymmetry is due to collateralization, vascular reconfiguration, or merely caused by prolonged ATT.

One potential explanation for sCoV’s superior sensitivity compared to CBF is that in asymptomatic carotid occlusion patients CBF remains relatively stable whereas the vascular supply undergoes changes such as collateralization and reconfiguration. We speculate that the sCoV reflects these vascular supply changes while the mean ASL signal reflects changes in tissue perfusion. If this is indeed the case, the sCoV, i.e., the ASL label distribution, may provide complementary information about the patient’s cerebrovascular status, which would aid in clinical decision-making, especially since the recruitment of collateral pathways is considered an important predictor for carotid disease and intervention outcome [4]. While mean CBF is relatively insensitive to ATT variations in large ROIs, using smaller and more distal ROIs can render CBF more sensitive to changes in vascular supply. Using MR angiography, Hendrikse et al. have shown higher presence of collateral flow in the anterior part of the circle of Willis in asymptomatic patients compared to symptomatic patients and healthy controls [26]. These findings are in agreement with the results presented here and underscore the potential added value of sCoV for assessing vascular reconfiguration in asymptomatic patients. Future research is needed to investigate whether sCoV can be used in tandem with other parameters to probe specific mechanisms underlying the brain's response to carotid occlusion.

Another potential explanation for our findings is an indirect association between the spatial CoV and the side of occlusion. Patients with internal carotid disease are likely to have a degree of both large and small vessel disease [27], which have shown to be associated with the spatial CoV [28–31]. It is likely that these factors have contributed to the sCoV and CBF measures in our cohort.

While this is the first study to quantify the presence of intra-vascular label in cerebrovascular disease, results are in agreement with the qualitative results from previous studies. Intra-vascular label in ASL has been used to visually detect the presence of ICA and MCA occlusion [32,33], the site of ICA occlusion [34], and the presence of collateral flow [35]. The side of occlusion has been detected visually with high sensitivity, 94% in the Kato et al. [33] and 88% in the Tada et al. study [32]. It is important to emphasize that these studies have included only symptomatic patients. Our findings in asymptomatic patients cannot be readily extrapolated to symptomatic patients as they may lack collateral flow which would likely cause CBF asymmetries without necessarily increasing the asymmetry of sCoV. A recent study that used ASL to detect the presence of collaterals in asymptomatic patients with MCA occlusion also found no inter-hemispheric difference in CBF [19]. This finding is in agreement with the results presented here from patients with ICA occlusion thus underlining the potential benefit of using sCoV for assessing the presence of collateral flow. While ipsilateral sCoV is expected to increase with a higher collateral score, this relation needs to be confirmed by comparing it with established measures of collaterals such as the mCTA collateral score. The behavior of sCoV may differ between symptomatic and asymptomatic patients, and therefore should be evaluated over a complete range of collateral scores.

The main advantage of using sCoV from single-delay ASL images for assessing ATT is that unlike the multi-PLD method, which provides a more direct measurement of ATT, the computation of sCoV from single-PLD data does not require additional clinical scanning time. Furthermore, in contrast to the currently used visual inspection, which is qualitative in nature and affected by inter-rater variability, the sCoV method presented here is fully automated and has the key advantage of being quantitative, thus enabling the measurement of the effects of inter- and intra-patient differences in delayed arrival times.

The sCoV measurement of this study has several limitations. First, the knowledge of the Circle-of-Willis configuration is not included in our population. Certain normal variants of Circle-of-Willis, such as the unilateral fetal-type, can cause CBF asymmetry [36], and can potentially affect the sCoV asymmetry as well. However, any bias in our results is expected to be minimal since significant CBF asymmetries from normal-variants are mainly found in the posterior circulation [36]. Investigating the use of sCoV in different Circle-of-Willis configurations remains a subject for future research.

A second potential limitation is the absence of an M0 scan for obtaining absolute CBF values and to correct for coil inhomogeneity bias and obtain absolute CBF values. Lack of M0 should not have affected our results as our asymmetry metrics do not rely on absolute quantification. Moreover, we did not observe any spatial inhomogeneity in the ASL control images (data not shown). There was a sex imbalance in the study population. While CBF and sCoV baseline values indeed correlate with sex [16], sex is not expected to influence the asymmetry index of sCoV. It should also be acknowledged that the added value of sCoV as cerebrovascular marker is dependent on the PLD. For this study, PLD ranged, slice-wise, from 1200 to 2025 ms, with inferior slices having lower PLD values than typically used for such population [5]. This choice likely compromised the accuracy of the CBF estimation, especially in the superior slices, and benefited the vascular information captured by sCoV. However, considering that there was no effect of slice location in the observed asymmetry of sCoV, we anticipate that the sCoV parameter will remain useful for acquisitions with longer PLDs, particularly since collateralization and reconfiguration can increase the ATT by 1–2 seconds [35]. Nevertheless, the dependency of sCoV on PLD remains to be evaluated.

Also, while this study showed the utility of sCoV for predicting the side of occlusion in all but one asymptomatic patient, the lack of a control group makes it difficult to establish and validate a cut-off value for sCoV AI reflecting “normal” asymmetry.

Lastly, the distribution of the degree of contralateral stenosis was too narrow to investigate the relationship between sCoV and contralateral stenosis. Since this study was focused on comparing the sensitivity of sCoV with that of CBF for detecting differences in flow between hemispheres, the same degree of collateral flow would be in effect regardless of the measurement method. Future research is needed to investigate this relationship.

## Conclusions

The sCoV parameter was introduced as a quantitative measure of intravascular signal in ASL imaging of carotid occlusive disease. Results showed that sCoV was stronger correlated with the side of occlusion than mean CBF. These proof-of-principle findings support a potential clinical role for sCoV as a proxy for delayed label arrival resulting from collateral perfusion and vascular reconfiguration associated with cerebrovascular disease.
